# Analysis of factors influencing postoperative refractive status in patients with high myopia undergoing combined surgery for vitreous opacity and cataract

**DOI:** 10.1371/journal.pone.0344563

**Published:** 2026-03-06

**Authors:** Yajun Wu, Junrong Liang, Jiasong Yang, Xiaolin Xie, Hua Fan, Wensheng Li

**Affiliations:** 1 Nanchang Aier eye hospital, Nanchang, Jiangxi, China; 2 Hunan Aier Eye Research Institute, Changsha, Hunan, China; 3 Aier Academy of Ophthalmology, Central South University, Changsha, Hunan, China; 4 Shanxi Aier eye hospital, Taiyuan, Shanxi, China,; 5 Shanghai Aier eye hospital, Shanghai, China; 6 Shanghai Aier eye institute, Shanghai, China,; 7 Aier Eye Hospital, Jinan University, Guangdong, China; Alexandria University Faculty of Medicine, EGYPT

## Abstract

**Aim:**

To observe the postoperative efficacy and factors affecting refractive status following combined vitrectomy and cataract surgery for high myopia (HM) with cataract and vitreous opacities, and compare the differences in refractive status between eyes with and without posterior capsulotomy.

**Method:**

Retrospective case study, a total of 57 patients (81 eyes) diagnosed with HM complicated with cataracts and vitreous opacity at Shanghai Aier Eye Hospital between 01/01/2022 and 01/01/2025.were included in the study, who underwent pars plana vitrectomy (PPV) combined with cataract phacoemulsification and intraocular lens (IOL) implantation. Preoperative examinations included best corrected visual acuity (BCVA), IOL-master, and Pentacam, with IOL power calculated using the Barrett formula. Postoperative follow-up was conducted at 3 months, including BCVA and refraction assessment. Besides, compared and analyzed the refractive status of eyeballs with and without posterior capsule incision during surgery(60 eyes with posterior capsulotomy and 21 eyes with posterior capsule preservation).

**Results:**

The mean axial length (AL) of patients’ eyes was 29.77 ± 2.55 mm, and the mean preoperative BCVA was 0.64 ± 0.60 (LogMAR). Three months postoperatively, the mean BCVA improved to 0.40 ± 0.43 (LogMAR), showing a statistically significant improvement (P < 0.05). The mean postoperative spherical equivalent (SE) was −2.83 ± 1.57 degree (D), with an average deviation of −0.23 ± 0.55 (D) from the target refraction. The refractive deviation of all eyes was −0.23 ± 0.55D, and −0.20 ± 0.56D in the posterior capsulotomy group, which was less than −0.30 ± 0.53D in the posterior capsule preservation group compared with the target refractive deviation(p = 0.49). In addition, preoperative SE, postoperative SE, and reserved diopter were significantly correlated with postoperative refractive deviation (r = 0.37,p = 0.0007;r = 0.24,p = 0.03;r = 0.61,p < 0.0001), while age and anterior chamber depth (ACD) were respectively correlated with refractive deviation (r = 0.16,p = 0.22; r = −0.10,p = 0.39).

**Conclusion:**

Combined surgery for HM with cataract and vitreous opacities improves postoperative vision. The mean deviation between reserved refraction and target refraction is less than 0.5 D. Refractive deviation is significantly correlated with preoperative/postoperative SE and reserved degree, may have a positive correlation with age,and may be negatively correlated with ACD. Posterior cystectomy may also be one of the influencing factors in reducing refractive deviation.

## Introduction

High myopia (HM) has become one of the major public health problems worldwide. Especially in Asia, the prevalence of HM among young adults is as high as 10–20% [1,2]. HM can induce various ocular complications [3,4]. These complications have a significant impact on patients’ visual impairment. Ordinarily, patients with HM, whether they have cataracts or severe vitreous opacity, usually require surgical treatment [5].

When HM patients develop conditions such as retinal holes, the vitreous body may undergo liquefaction and opacification. Severe vitreous opacification is difficult to treat effectively with neodymium:yttrium-aluminum-garnet (Nd:YAG) laser alone, and pars plana vitrectomy (PPV) is usually required [6,7]. In addition to concurrent vitreous opacification, HM patients also have a significantly increased risk of developing cataracts [8]. Furthermore, the refractive status of HM patients after cataract surgery is often more difficult to control, with refractive deviation being relatively common [9], and the incidence of intraoperative and postoperative complications is also higher than in the general population [10–13]. Literature indicates that HM itself and a previous history of PPV are both significantly associated with the occurrence of refractive deviation after cataract surgery [14]. In fact, HM patients who undergo simple cataract surgery have a higher risk of developing vitreoretinal diseases [15]; conversely, patients with vitreoretinal diseases who undergo simple PPV have an increased risk of developing cataracts (especially posterior capsular opacification, PCO) [16,17].When high-risk cataract patients are complicated with both cataract and vitreous opacity, simultaneous management of cataract and retinal lesions is required. Sequential surgeries not only lead to more complications [11,15] but also impose dual physical, psychological, and economic burdens on patients. Therefore, we adopted PPV combined with phacoemulsification and intraocular lens (IOL) implantation for the treatment of high-risk cataract patients with vitreous opacity. Oh J et al [17] conducted a study on complex cataract patients without severe posterior segment lesions and found that patients achieved significant visual improvement 3 months after surgery. In addition, this combined surgery can reduce complications associated with standalone PPV or standalone cataract surgery, avoid multiple surgeries for patients, significantly improve the visibility of posterior segment tissues during PPV, and prevent accidental lens damage [18]. Awidi AA et al [19] compared the efficacy of cataract surgery after PPV, PPV after cataract surgery, and combined surgery, and found that both combined surgery and sequential surgery significantly improved patients’ visual acuity with similar therapeutic effects; furthermore, combined surgery reduces one surgical procedure, avoids complications such as cataract progression after standalone PPV, and is more suitable for patients over 60 years old [19]. Additionally, in 2023, our team first reported 3 cases of extreme HM (>-30D) patients who underwent combined surgery, all of whom achieved significant postoperative visual improvement and complete resolution of vitreous opacity symptoms [20]. We believe that phacoemulsification combined with PPV is an ideal treatment option for high myopia complicated with cataract and vitreous opacity, but due to the small sample size at that time, it was only published as a case report.

Currently, patients with HM complicated by cataract and vitreous opacity face notable clinical challenges: sequential surgeries are associated with increased complication risks, multiple procedural burdens, and higher postoperative refractive deviation rates, which severely impact visual outcomes. Despite the growing application of phacoemulsification combined with PPV for such HM patients, relevant research remains limited—particularly critical gaps exist regarding whether intraoperative posterior capsulotomy affects refractive stability. To address these gaps, this study retrospectively analyzed clinical data of HM patients with cataract and vitreous opacity who underwent the combined surgery, aiming to: 1.Identify the key factors contributing to postoperative refractive deviation; 2.clarify the impact of intraoperative posterior capsulotomy on postoperative refractive status. The findings are expected to provide evidence-based guidance for optimizing surgical strategies and improving the accuracy of refractive prediction in this high-risk cohort.

## Method

### Data access and ethics

Data Access: The medical records, ophthalmic examination results (including IOL-master, Pentacam, BCVA, and refraction data), and surgical records of the included patients were accessed for research analysis between [01/02/2025] and [15/04/2025]. This study was approved by the Ethics Committee of Shanghai Aier Eye Hospital (SHAIER2025YN013) and followed the principles of the Helsinki Declaration.

### Research subjects

This retrospective study included 57 patients diagnosed with HM and complicated with cataract and vitreous opacity who were hospitalized at Shanghai Aier Eye Hospital from 01/01/2022–01/01/2025, involving 81 eyes. All patients underwent PPV combined with phacoemulsification and intraocular lens implantation under local anesthesia. 60 eyes were selected for intraoperative posterior capsulotomy (After phacoemulsification, aspiration, and cortical polishing of cataracts, if posterior capsular opacity or residual tiny cortical fragments that could not be completely removed were still observed, posterior capsulotomy was performed after IOL implantation and adjustment to the optimal position. This was to reduce the risk of postoperative PCO and provide a clearer surgical field of view for subsequent vitrectomy), while the remaining 21 eyes had their posterior capsules left intact. All patients signed the informed consent form for surgery approved by Shanghai aier hospital and underwent combined surgery. Inclusion criteria: 1. Patients with HM who received combined cataract and vitrectomy surgery at Shanghai Aiye Eye Hospital; 2. Patients diagnosed with HM (axial length [AL] ≥ 26 mm or refractive error [RE] ≤ -6D), accompanied by cataract and vitreous opacity; 3. Patients with severe cataract that affects daily life and the operation of fundus surgery; 4. Moderate to severe vitreous opacity, ineffective with conservative treatment or Nd:YAG laser treatment,and floaters persisted in the visual field, affecting visual quality and daily activities; 5. No complex serious lesions in the fundus (such as multiple retinal holes accompanied by retinal detachment, macular hole-related retinal detachment, severe proliferative traction, etc., requiring silicone oil filling for fundus diseases), and only requiring simple PPV resection; 6. Patients without previous eye trauma or undergoing previous ophthalmic surgery; 7. Patients with intact zonular fibers of the lens and intact posterior capsule of the lens; 8. Patients with stable general condition and requiring combined surgery. Exclusion criteria: 1. Patients unwilling to undergo combined surgery; 2. Patients without high myopia (AL < 26 mm/RE > -6D); 3. Patients with other ocular diseases such as glaucoma and uveitis in the operated eye; 4. Patients with ocular trauma or previous surgical history in the operated eye; 5. Patients with lens subluxation; 6. Patients with severe fundus lesions requiring silicone oil or gas tamponade during surgery.

All the included patients were those without obvious macular lesions (such as macular hemorrhage, exudation, holes, CNV and macular schisis). Peripheral fundus lesions are limited to complex conditions such as simple lattice degeneration, atrophic holes, no large retinal holes, multiple retinal holes, and tractional retinal detachment.

Besides, all patients in this study were implanted with in-the-bag monofocal IOL (Model: MA60AC, Alcon Laboratories, Inc., USA). Preoperatively, the tension and integrity of the lens zonular fibers were evaluated using slit-lamp biomicroscopy, ocular B-ultrasound, and the Pentacam three-dimensional anterior segment analysis system. It was confirmed that all included cases had no zonular relaxation, rupture, or weakness, so no capsular tension ring (CTR) was used. During the surgery, continuous curvilinear capsulorhexis combined with in-the-bag IOL implantation was performed to ensure stable fixation of the IOL within the capsular bag.

### Ophthalmic examination

All patients underwent IOL-master 500 measurement of the AL before surgery, calculation of the intraocular lens power using the Berette Universa II formula, optometry using a comprehensive optometry instrument, and routine examinations including three-dimensional anterior segment analysis (Pentacam) for anterior chamber depth (ACD), dilated fundus examination, BCVA measurement, and slit lamp examination. Three months after surgery, they underwent BCVA and comprehensive optometry examinations in the outpatient department, with the results recorded as SE (SE = spherical diopter +1/2 cylinder diopter)

### Surgical method

All surgeries were performed by the same surgeon.

The patient was placed in a supine position for oxygen inhalation. Operated eyeunderwent surface anesthesia, conjunctival sac irrigation, and routine surgical field disinfection and draping. 2. Under electrocardiographic monitoring, a mixture of lidocaine hydrochloride and bupivacaine (about 4 ml) was administered for retrobulbar nerve block anesthesia. After successful anesthesia, an eyelid opener was placed. 3. Under a microscope, an auxiliary incision was made at the 2 o'clock position. Viscoelastic agent was injected into the anterior chamber. A 2.2.mm clear corneal incision was made at the 10 o'clock position, followed by circular capsulorhexis, hydrodissection, and hydrostratification. The lens nucleus was aspirated using phacoemulsification, and residual cortex was aspirated along with the removal of residual sodium hyaluronate. 4. Three channels were established, and a wide-angle intraocular lens (IOL) was placed to remove turbid vitreous. The retina was flattened, and the degenerative area or hole of the retina was sealed using laser. 5. Viscoelastic agentwas injected into the anterior chamber, and a single-vision intraocular lens with an appropriate power for the patient was implanted in the capsule. Residual sodium hyaluronate was removed, and the incision was sealed with water. 6. In the posterior capsule removal group, the central posterior lens capsule was removed, and the three channels were closed. 7. After the surgery, the operated eye was coated with tobramycin and dexamethasone ophthalmic ointment, and the patient was returned to the ward. The operation went smoothly and no complications such as iatrogenic retinal perforation occurred in any of the patients.

All patients underwent mydriatic fundus examination combined with ultra-wide-angle fundus photography before the operation to accurately locate the position and extent of the lattice degeneration area and retinal holes, and the results were recorded in the surgical plan. 2. After vitrectomy is completed, the lesion area is reconfirmed through the wide-angle fundus observation system. Retinal laser photocoagulation is performed on the grid-like degeneration area to ensure that the photocoagulation spots are continuous and uniform. For isolated atrophic holes, 2–3 rows of laser photocoagulation sealing should be performed around the edge of the hole to avoid missing the lesion area. 3. If a small amount of fluid was found around the tiny holes during the operation, the retina was flattened by filling the eye with balanced salt solution before photocoagulation was performed to ensure the photocoagulation effect. None of the patients required additional silicone oil or gas filling due to fundus lesions.

### Statistical analysis

Statistical analysis was performed using SPSS 25.0 (IBM Corp., Armonk, NY, USA). All results are presented as mean ± standard deviation (SD). Outliers were identified using the ROUT method, and the Shapiro-Wilk test was used to assess whether the data followed a normal distribution. Levene's test was employed to assess homogeneity of variance. For data that did not meet the assumption of homogeneity of variance, Welch's method was used for correction. Paired t-tests were used to compare data for the same indicator before and after surgery, while unpaired t-tests were used to compare the capsule-cutting group with the non-capsule-cutting group. The Wilcoxon test was applied to data that did not follow a normal distribution. Spearman's correlation analysis was used to analyze the correlation between various indicators. P ≤ 0.05 was considered statistically significant.Statistical methods

## Result

### Basic information of patients and results of preoperative ophthalmic parameters

A total of 57 patients (81 eyes) were included, consisting of 25 females and 32 males, with 43 right eyes and 38 left eyes. The mean age of all patients was 57.12 ± 12.89 years, the mean AL was 29.77 ± 2.55 mm, the mean ACD was 3.36 ± 0.32 mm, the preoperative BCVA was 0.64 ± 0.60LogMAR, and the preoperative SE was −7.20 ± 3.59 D;

The posterior capsulotomy group included 42 patients (17 males and 25 females, 60 eyes total), with a mean age of 56.45 ± 12.97 years, a mean AL of 29.95 ± 2.63 mm, a mean ACD of 3.34 ± 0.36 mm, a preoperative BCVA of 0.60 ± 0.58 LogMAR, and a preoperative SE of −7.49 ± 3.70 D;

The posterior capsule preservation group included 15 patients (8 males and 7 females, 21 eyes total), with a mean age of 59.00 ± 12.93 years, a mean AL of 29.27 ± 2.35 mm, a mean ACD of 3.42 ± 0.15 mm, a preoperative BCVA of 0.65 ± 0.62 LogMAR, and a preoperative SE of −6.37 ± 3.20 D, refer to Table 1 for details.

**Table 1:** Basic information and ocular characteristics.

Parameters	All	Posterior capsulotomy group	Posterior capsule preservation group
**Number of patients**	57	42(74%)	15(26%)
**Number of eyeballs**	81	60(74%)	21(26%)
**right eye**	43(53%)	32(53%)	11(52%)
			left eye	38(47%)	28(47%)	10(48%)
**Gender**	25M/32F	17M/25F	8M/7F.		
**male**	25(44%)	17(40%)	8(53%)
	**female**	32(56%)	25(60%)	7(47%)
**Age(mean±SD)**		57.12 ± 12.89	56.45.12 ± 12.97	59.00 ± 12.93
**AL(mean±SD)mm**	29.77 ± 2.55	29.95 ± 2.62	29.27 ± 2.35
**Preoperative SE**	−7.20 ± 3.59	−7.49 ± 3.70	−6.37 ± 3.20
**Preoperative BCVA (LogMAR)**	0.64 ± 0.60	0.60 ± 0.58	0.65 ± 0.62
**ACD (mm)**	3.36 ± 0.32	3.34 ± 0.36	3.42 ± 0.15

ACD, anterior chamber depth; BCVA, best corrected visual acuity; SE, spherical equivalent; M, male; F, female.

**Fig 1:**
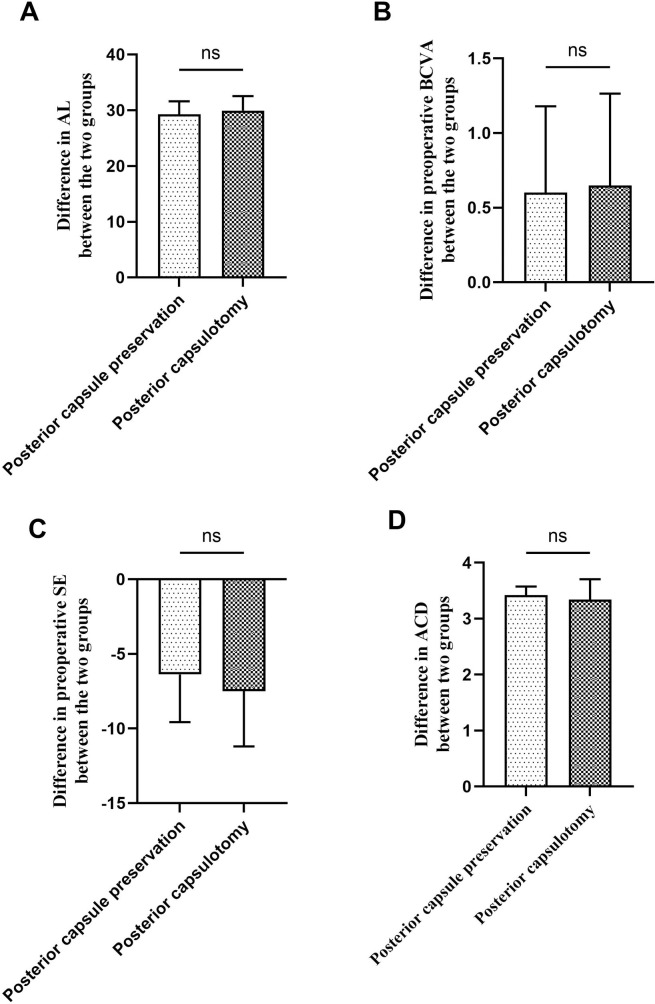
Comparison of preoperative ophthalmic parameters between the posterior capsulotomy group and the posterior capsule preservation group. A. Comparison of AL between the two groups; B.Comparison of preoperative BCVA between the two groups; C.Comparison of preoperative SE between the two groups; D.Comparison of ACD between the two groups; AL.axial length; ACD, anterior chamber depth; BCVA,best corrected visual acuity; SE,spherical equivalent.

Comparisons between the two groups showed no statistically significant differences in AL (p = 0.30, t = 1.04, Fig 1A), preoperative BCVA (p = 0.75, t = 0.31, Fig 1B), preoperative SE (p = 0.22, t = 1.23, Fig 1C), ACD (p = 0.35, t = 0.93, Fig 1D), or age (p = 0.52, t = 0.65).

### The therapeutic effect of combined surgery after 3 months follow-up

The average reserved degree of all ocular lenses was −2.60 ± 1.34 D, the posterior capsulotomy group was −2.60 ± 1.29 D, and the posterior capsule preservation group was −2.62 ± 1.50 D. There was no statistically significant difference between the two groups (p = 0.70). The refractive deviation of all eyes was −0.23 ± 0.55D, that of the posterior capsulotomy group was −0.20 ± 0.56 D, and that of the posterior capsule preservation group was −0.30 ± 0.53 D There was no statistically significant difference between the two groups (p = 0.49,t = 0.70).

**Fig 2:**
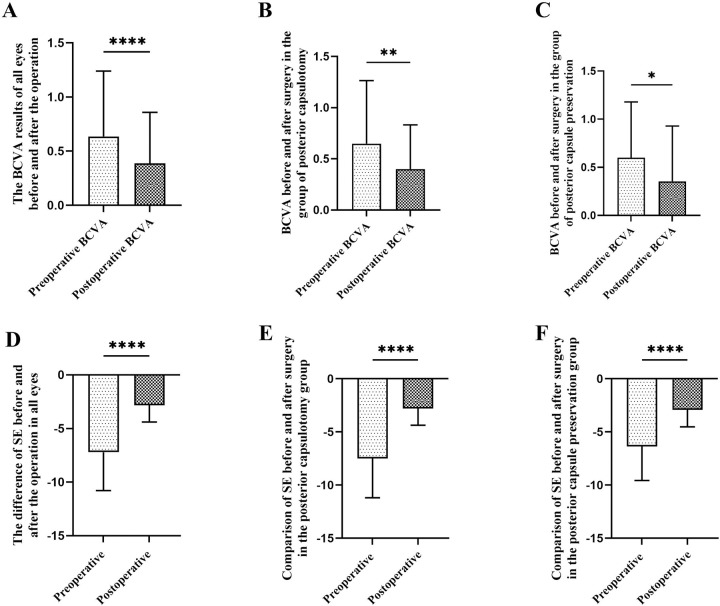
Comparison of BCVA and SE at 3 months postoperatively with preoperative results. A.Comparison of logMAR BCVA before and after surgery for all eyes; B.Comparison of logMAR BCVA before and after surgery in the posterior capsulotomy group; C.Comparison of logMAR BCVA before and after surgery in the posterior capsule preservation group; D.Comparison of postoperative SE before and after surgery for all eyes; E.Comparison of postoperative SE before and after surgery in the posterior capsulotomy group; F.Comparison of postoperative SE before and after surgery in the posterior capsule preservation group; BCVA,best corrected visual acuity; SE,spherical equivalent; *p < 0.05, **p < 0.01，****p < 0.0001.

The BCVA (LogMAR) of all eyes increased from 0.64 ± 0.60 to 0.40 ± 0.43 (p < 0.0001, Fig 2A), and the BCVA of the posterior capsulotomy group increased from 0.60 ± 0.58 to 0.40 ± 0.43 (p = 0.00, Fig 2B); The BCVA of the posterior capsule preservation group increased from 0.65 ± 0.62 to 0.35 ± 0.58 (p = 0.045,t = 2.14, Fig 2C); Postoperative SE: All eyes recovered from −7.20 ± 3.59 D to −2.83 ± 1.57 D (p < 0.0001,t = 14.49, Fig 2D), the posterior capsulotomy group recovered from −7.49 ± 3.70 D to −2.80 ± 1.57 D (p < 0.0001,t = 12.83, Fig 2E), the posterior capsule preservation group recovered from −6.37 ± 3.20 to −2.92 ± 1.61 (p < 0.0001,t = 7.35, Fig 2F).Details are shown in Table 1 and 2.

**Table 2:** Postoperative ophthalmic parameter results.

Ophthalmic parameters	All	Posterior capsulotomy group	Posterior capsule preservation group
**Reserved degree (D)**	−2.60 ± 1.34	−2.60 ± 1.29	−2.62 ± 1.50
**Postoperative BCVA (LogMAR)**	0.40 ± 0.43	0.40 ± 0.43	0.35 ± 0.58
**Postoperative SE**	−2.83 ± 1.57	−2.80 ± 1.57	−2.92 ± 1.61
**Refractive deviation (D)**	−0.23 ± 0.55	−0.20 ± 0.56	−0.30 ± 0.53

BCVA, best corrected visual acuity; SE, spherical equival.

In addition, the differences in reserved degree (p = 0.96, t = 0.05), refractive deviation (p = 0.49, t = 0.70), postoperative BCVA (p = 0.23), and postoperative SE (p = 0.77, t = 0.29) between the posterior capsule preservation group and the posterior capsule preservation group were not statistically significant.

### Correlation analysis among various parameters

**Fig 3:**
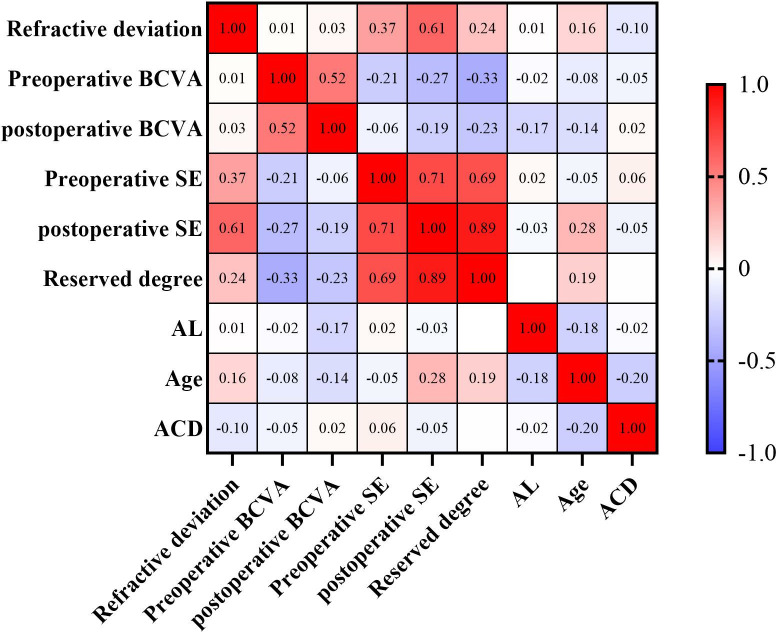
Heat map of correlation analysis among various ophthalmic parameters. AL.axial length; ACD, anterior chamber depth; BCVA,best corrected visual acuity; SE,spherical equivalent; Blue represents the negative correlation, red for positive correlation, the closer the correlation coefficient of the absolute value of 1 represents the greater the correlation.

The Spearman r between refractive deviation and preoperative SE was 0.37 (p = 0.0007); the Spearman r with postoperative SE was 0.61 (p < 0.0001); and the Spearman r with reserved degree was 0.24 (p = 0.03), all showing statistically significant positive correlations. In addition, the Spearman r between refractive deviation and age was 0.16(p = 0.22), indicating a positive correlation; the Spearman r with ACD was −0.10(p = 0.39), indicating a negative correlation, but neither correlation was statistically significant, as shown in Fig 3 and Table 3.

**Table 3:** Spearman correlation analysis results among various ophthalmic parameters.

P value	Refractive deviation	Preoperative BCVA	postoperative BCVA	Preoperative SE	postoperative SE	Reserved degree	AL	Age	ACD
**Refractive deviation**		0.92	0.80	0.0006***	****	0.03*	0.95	0.22	0.39
**Preoperative BCVA**	0.92		****	0.07	0.015*	0.002**	0.86	0.55	0.66
**postoperative BCVA**	0.80	****		0.62	0.09	0.04*	0.14	0.31	0.83
**Preoperative SE**	0.0006***	0.07	0.62		****	****	0.82	0.74	0.63
**postoperative SE**	****	0.01	0.09	****		****	0.80	0.04*	0.65
**Reserved degree**	0.02*	0.002**	0.04*	****	****		0.98	0.15	0.98
**AL**	0.95	0.86	0.13	0.81	0.80	0.98		0.18	0.88
**Age**	0.22	0.55	0.31	0.74	0.04*	0.15	0.18		0.15
**ACD**	0.39	0.66	0.83	0.63	0.65	0.98	0.88	0.15	

AL, axial length; ACD, anterior chamber depth; BCVA, best corrected visual acuity; SE, spherical equivalent; *p < 0.05, **p < 0.01，***p < 0.001, ****p < 0.0001.

## Discussion

Up to now, there have been relevant reports on combined surgery internationally. Vatavuk Z et al [21]. reported on patients with advanced diabetic retinopathy treated with combined surgery and found that 80% of the patients had good postoperative vision recovery, while a small number of other patients developed complications such as recurrent vitreous hemorrhage and glaucoma, they confirmed the effective efficacy of combined surgery for patients with advanced diabetic retinopathy. Furthermore, Batman C et al [22]. reported the combined surgery for the removal of intraocular foreign bodies with lens opacity due to ocular trauma, the intraocular lenses of all patients were stable after the operation, and the BCVA of 74% of the patients improved after the operation without significant complications, confirming the good efficacy of the combined surgery in ocular trauma. Besides, koushan K et al [23]. compared the visual acuity improvement and corneal endothelial cell density (ECD) changes of the two groups after 3 months in order to evaluate the impact of combined surgery and PPV alone on patients, they found that although the postoperative ECD of patients with combined surgery was slightly lower than that of patients with PPV alone (no statistical difference), the visual acuity of patients with combined surgery was significantly better than that of patients with PPV alone, which showed that combined surgery had significant advantages in improving the visual acuity of patients and was a recognized surgical method.

Similar to other literature on combined surgery, our study also found that combined surgery can significantly improve the vision of patients with HM combined with cataract and vitreous opacity. Unlike other studies, we explored the influencing factors of refractive status in HM patients after combined surgery, especially the influencing factors of refractive deviation, and evaluated whether intraoperative and postoperative capsulectomy was a key factor for changes in postoperative refractive status. We found that although the difference was not statistically significant, the refractive deviation of the posterior capsulotomy group was slightly smaller than that of the posterior capsulotomy group (the refractive deviation of the two groups were −0.20 ± 0.56 D, −0.30 ± 0.53 D, respectively). Moreover, most patients (74%) underwent posterior capsulotomy, and we reserved the posterior capsule for a few patients (26%), the reason is that the residual lens epithelial cells in cataract surgery patients are difficult to completely remove, and the lens epithelial cells undergo proliferation, migration, and epithelial mesenchymal transition, ultimately leading to the PCO [24]. Therefore, we directly remove the posterior capsule during surgery to avoid the occurrence of PCO. Although the posterior capsule can be cut by Nd:YAG laser in the later stage [25,26], but this is an additional invasive procedure that requires patients to spend time and effort on treatment again. Otherwise, we are not sure whether posterior capsulectomy will significantly affect the improvement of visual acuity, so we retained the posterior capsule of some patients, hoping to evaluate the postoperative difference between the two groups. Finally, our results confirmed that posterior capsulotomy combined with surgery can not only improve the visual acuity of patients, but also the refractive deviation may be less than that of patients without posterior capsulotomy, which is a favorable surgical method for HM patients with cataract and vitreous opacity.

Although the goal of cataract surgery is to select an IOL with an appropriate power for patients to achieve precise postoperative refractive outcomes, it remains challenging for patients with HM to attain the target refractive state postoperatively. Refractive drift is relatively common in HM patients after cataract surgery, which may be associated with factors such as the selection of IOL power calculation formulas, patient gender, and age [27–29]. Arens S et al. [29] reported the presence of refractive errors following cataract surgery, noting that patients experienced myopic drift with an overall average deviation of −0.39 D from the target diopter, and hyperopic drift was more pronounced in elderly patients. In the present study, the refractive deviation in the posterior capsulotomy group was slightly lower than that in the posterior capsule preservation group (−0.20 ± 0.56 D vs. −0.30 ± 0.53 D, respectively), and there was myopic drift relative to the target refractive state of the IOL postoperatively (drift degree < −0.5 D). Additionally, a significant positive correlation was found between the degree of refractive deviation and preoperative SE. We hypothesize that a higher preoperative myopic degree (more negative SE) in HM patients is often accompanied by more marked axial elongation, posterior staphyloma formation, and intraocular structural deformation. These anatomical abnormalities can lead to minor errors in biometric measurements (e.g., axial length, corneal curvature), and IOL power calculation (using the Barrett formula in this study) is sensitive to such anatomical deviations, ultimately resulting in increased postoperative refractive deviation. Furthermore, the lens nucleus of HM patients may be harder, and the slight disturbance to the intraocular environment during phacoemulsification may indirectly affect refractive stability, exerting a synergistic effect with the preoperative high myopic state. On the other hand, although no statistically significant correlation was observed between age and refractive deviation in this study, Fig 3 shows a positive correlation trend, indicating that the older the patient, the greater the refractive deviation. Specifically, when the refractive deviation is positive, older patients exhibit more severe hyperopic drift, which is consistent with the findings of Arens S et al. [29]. We speculate that with advancing age, HM patients experience decreased scleral elasticity and weakened ciliary muscle function, which may slightly reduce the stability of postoperative intraocular structures (e.g., the capsular bag-IOL complex), thereby increasing the risk of mild hyperopic drift. However, the specific mechanism requires further verification through larger-sample cohort studies.

In addition, postoperative SE and reserved refractive power were also positively correlated with the degree of refractive deviation. Typically, in cataract surgery, an appropriate IOL is selected to achieve a target refractive error of 0.00 D for the dominant eye, while different refractive errors are reserved for the non-dominant eye [30]. However, for HM patients with cataracts, it is more difficult to achieve the target refractive state postoperatively, and refractive deviation is relatively more common [31]. Since HM patients have been in a myopic state for a long time, retaining a reasonable degree of myopia can optimize their functional vision, provide high-quality uncorrected near vision, and meet their daily living needs. Therefore, it is usually necessary to reserve a certain degree of myopia for HM patients and attempt to control the refractive error within ±0.50 D [32,33]. We hypothesize that the achievement of reserved refractive power relies on the precise matching of IOL power and the stability of postoperative intraocular structures. Due to the larger intraocular space and individual differences in zonular tension among HM patients, even if the IOL is implanted in the capsular bag and adjusted to the optimal position, minor postoperative IOL positional drift (e.g., anterior-posterior displacement) may still occur. Such subtle changes directly lead to deviations between the actual postoperative SE and the target reserved refractive power. In summary, the results of this study indicate that preoperative SE and reserved refractive power are the main factors influencing postoperative refractive deviation, age may also be one of the influencing factors, and the degree of refractive deviation in the intraoperative posterior capsulotomy group is smaller than that in the posterior capsule preservation group. Therefore, we believe that posterior capsulotomy can not only avoid the occurrence of PCO but also may reduce the degree of myopic drift, although this speculation requires further verification with a larger sample size in future studies. Moverover, this study found a negative correlation trend between ACD and refractive deviation (r = −0.10, p = 0.39). Although not statistically significant, it still holds clinical reference value. The potential mechanism may be that ACD is a core parameter for IOL power calculation. Although the Barrett formula used in this study has incorporated the ACD variable, axial elongation and scleral expansion in patients with high myopia may lead to minor errors in ACD measurement. When the actual ACD is greater than the measured value, it is likely to result in an underestimation of the calculated IOL power and postoperative myopic drift; conversely, it may lead to hyperopic drift. This inverse association between measurement error and refractive deviation may form the basis of the observed negative correlation trend.

However, our study has several limitations. First, selection bias is inevitable as the decision for intraoperative posterior capsulotomy was based on real-time surgical findings (e.g., posterior capsular opacity or residual cortex) rather than random allocation. Second, the 3-month short-term follow-upis insufficient to evaluate long-term refractive stability and late changes in the IOL–capsular complex in HM eyes, limiting the generalizability of our findings to long-term outcomes. Third, the results are only applicable to “relatively stable” HM patients with intact zonular function and no complex fundus pathology, and cannot be extended to those with severe retinal lesions, extremely long AL, or suspected zonular fragility. Future prospective randomized controlled studies with larger sample sizes and extended follow-up (≥1 year) are needed to minimize bias and validate the long-term efficacy.

## Conclusion

PPV combined with phacoemulsification and IOL implantation can effectively improve the refractive status of patients with HM complicated by cataracts and vitreous opacity. The occurrence of myopia drift deviating from the target refractive power after surgery is positively correlated with preoperative SE and reserved degree, and may be related to age, ACD and whether the posterior capsule is removed during surgery. Notably, due to the non-randomized grouping based on intraoperative findings, the potential influence of selection bias cannot be excluded when interpreting the association between posterior capsulotomy and refractive deviation.The younger the age, the greater the ACD, and the greater the degree of myopic deviation in patients with posterior capsule preservation, but more patients need to be included in the future to verify our conclusions.

## Supporting information

S1 Data Text. (XLSX)
